# Multi-channel multi-distance broadband near-infrared spectroscopy system to measure the spatial response of cellular oxygen metabolism and tissue oxygenation

**DOI:** 10.1364/BOE.7.004424

**Published:** 2016-10-05

**Authors:** Phong Phan, David Highton, Jonathan Lai, Martin Smith, Clare Elwell, Ilias Tachtsidis

**Affiliations:** 1 Department of Medical Physics and Biomedical Engineering, UCL, London WC1E 6BT, UK; 2 Neurocritical Care Unit, The National Hospital for Neurology and Neurosurgery, University College London Hospitals, Queen Square, London WC1N 3BG, UK

**Keywords:** (170.6510) Spectroscopy, tissue diagnostics, (170.3890) Medical optics instrumentation

## Abstract

We present a multi-channel, multi-distance broadband near-infrared spectroscopy (NIRS) system with the capability of measuring changes in haemoglobin concentrations (Δ[HbO_2_], Δ[HHb]), oxidation state of cytochrome-c-oxidase (Δ[oxCCO]) and tissue oxygen saturation (TOI) in the adult human brain. The main components of the instrument are two customized spectrographs and two light sources. Each spectrograph is lens-based to improve light throughput, has a grating enhanced to optimise reflection in the near-infrared (NIR) spectral region and uses a front illuminated cooled CCD camera (−70° C) with a square chip dimension of 12.3 x 12.3 mm (512 x 512 pixels). Each light source uses a 50W halogen bulb with a gold plated mirror to increase the intensity of the NIR light. Each light source was connected to a custom-built bifurcated fibre bundle to create two source fibre bundles (3.2 mm diameter each). Each spectrograph received light input from another custom-built fibre bundle comprised of six individual bundles (one with 0.6 mm diameter and the other five with 1.5 mm diameter). All fibre bundles were fixed on a 3D printed optode holder (two light sources x two fibre bundles each = four probes; and two spectrographs x six fibre bundles each = 12 probes) that allowed 24 multi-distance channels across the forehead (six channels at 20 mm, three channels at 30 mm and 15 channels at 35 mm) and six TOI measurements. We demonstrated the use of the system in a cohort of nine healthy adult volunteers during prefrontal cortex functional activation using the Stroop task. We have observed functional responses identified as significant increase in Δ[HbO_2_], decrease in Δ[HHb] and increase in Δ[oxCCO] in five channels (out of 12), that overlay the left and right dorsolateral prefrontal cortices. There was no observable TOI functional response and we have shown small variations in TOI across different sites within the same subject and within the same site across subjects.

## 1. Introduction

Near-infrared spectroscopy (NIRS) has for many years been used to investigate changes in haemodynamics and metabolism of various types of biological tissue, including the adult human brain [1–3]. To date, most commercial continuous wave (CW) functional NIRS (fNIRS) devices rely on dual wavelength approaches, limiting their capabilities to the measurements of concentration changes of oxygenated (Δ[HbO_2_]) and deoxygenated haemoglobins (Δ[HHb]) [4]. These measurements are not directly related to changes in metabolic activity during neuronal activation. An independent metabolic signal that is a direct marker of cellular oxygen metabolism may provide a platform to study the relationship between vascular haemodynamics and neuronal metabolism, as well as the balance between brain tissue oxygenation and utilisation in different scenarios. The broadband NIRS measurement of oxidised cytochrome-c-oxidase (Δ[oxCCO]) has great potential to be this candidate.

Cytochrome-c-oxidase (CCO) is the terminal enzyme in the mitochondrial electron transport chain, responsible for more than 95% of cellular oxygen metabolism for the synthesis of adenosine triphosphate. Measurement of the changes in the oxidation state of CCO can be used as a biomarker for cellular oxygen metabolism [5,6]. The difference in the absorption spectrum between the oxidised and the reduced species of CCO has distinct characteristics in the near-infrared region (NIR) (780 - 900 nm) with a broad peak at 830 nm and this can be used to resolve Δ[oxCCO]. Previously, we have extensively described an optimized NIRS approach for the detection of Δ[oxCCO] using broadband spectroscopy (780 – 900nm) [7]. This approach addresses specific challenges of detecting this chromophore: 1) its concentration is less than 10% of haemoglobin concentration and 2) risk of crosstalk artefact between chromophores with overlapping spectra [8–10]. A recent review by Bale and colleagues provides details on the spectral features of CCO, the methodology, analysis techniques as well as a summary of human brain studies regarding the NIRS measurement of cerebral oxCCO in both the healthy and injured brain [11].

Beyond the measurement of ∆[HbO_2_], ∆[HHb] and ∆[oxCCO], a more complex NIRS technique called spatially resolved spectroscopy (SRS) can be implemented to monitor the absolute level of oxygenation in tissue. SRS is an established technique using multiple source-detector distances to calculate the tissue oxygenation index (TOI), the ratio of oxygenated to total tissue haemoglobin, which can be used as a marker of the balance between tissue oxygen supply and demand [12,13]. This index has found initial success as a cerebral oxygenation monitor to guide haemodynamic and oxygenation management during cardiac surgery [14,15]. There is potential benefit in combining the measurements of ∆[HbO_2_], ∆[HHb] and ∆[oxCCO] with TOI to investigate the balance between cerebral oxygen delivery and utilisation in multiple clinical scenarios.

Investigations of the localisation of functional brain activity require recording of changes from multiple brain regions. In addition, certain brain injuries affect localised areas of the brain and it is of clinical importance to identify and monitor changes in those regions of interest. Moreover, previous investigation of Δ[oxCCO] using a dual channel system during functional activation by Kolyva et al. [16] suggested regional heterogeneity in the oxCCO response and this requires further investigation. However, to date, most systems that have the capability to measure changes in oxCCO signal and TOI have a limited number of channels, restricting measurements to only one or two locations. Table 1

**Table 1:** Brief summary of NIRS oxCCO measurement systems

NIRS systems	Number of wavelengths	Number of source(s)	Number of detector(s)	Number of simultaneous channels used	Multi-distance/ TOI
Duke 1 [17,18]	3	1	1	1	No/No
Duke 2 [19]	4	1	1	1	No/No
UCL 1 [20]	4	1	1	1	No/No
Keele 1 [21]	3	1	1	1	No/No
Keele 2 [22]	4	1	1	1	No/No
NIRO 1000 [23],	6	1	1	1	No/No
NIRO 500 [24]	4	1	1	1	No/No
NIRO 300 [13,25]	4	1	3	1	Yes/Yes
UCLn [26–28]	Broadband	1	1	1	No/No
UCLn–CYRIL [29]	Broadband	2	8	2	Yes/Yes
UCL Hybrid [30]	Broadband	2	8	2	Yes/Yes
Humboldt 1 [31]	Broadband	1	1	1	No/No
Humboldt 2 [10]	Broadband	1	4	4	No/No
Ryerson 1 [32]	Broadband	2	2	2	No/No

provides a brief summary of broadband NIRS systems with capability of measuring the ∆[oxCCO]. There is an unmet need to deliver a multi-channel broadband NIRS system to monitor ∆[oxCCO] and TOI.

The aim of this paper is to present a broadband NIRS system with the capability of measuring not only the haemodynamic changes, but also the oxCCO response, and TOI using 24 multi-distance channels across the prefrontal cortex of the adult brain. To demonstrate the capability of the system, we used it to monitor the prefrontal cortex during functional activation induced by the Stroop task; and we investigate the functional response of the oxCCO signal by identifying channels with significant change during the functional task. TOI values were recorded to demonstrate the capability of the system to monitor variations in oxygenation across multiple regions.

## 2. Methods

### 2.1 Instrumentation

Figure 1(a)

**Fig. 1:**
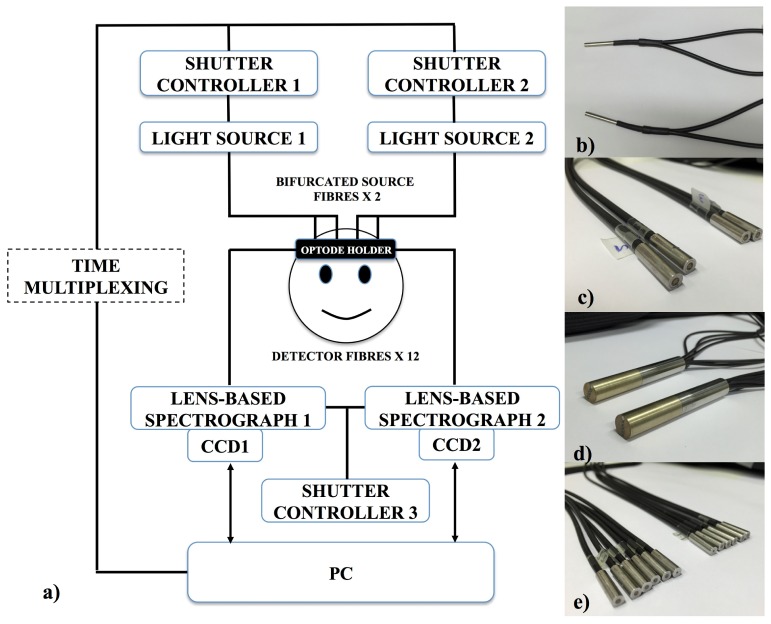
(a) Schematic showing all the components of the system. The system has two light sources, each has a solenoid shutter that is controlled by an electronic shutter controller with a time multiplexing mechanism. Light from the sources (filtered to 504nm – 1068nm) is directed to the subject by means of bifurcated optical fibre bundles, creating four source fibre bundles at the subject end. The diffused light from the subject head is collected using 12 detector fibre bundles branched from two fibre bundles, each of the two is connected to a spectrograph and a CCD camera. Both spectrographs are controlled by another electronic shutter controller. The system is run by LabVIEW software from a computer. (b) The common ends (attached to the light sources) of two bifurcated source fibre bundles. Bifurcation creates four individual source fibre bundles (c) at the subject end from two common bundles. (d) The detector fibre bundles at the spectrograph end. (e) The twelve individual detector fibre bundles which are branched from (d) and are attached to the subject.

shows the schematic of the multi-channel, multi-distance broadband NIRS system. It employs two light sources each with a gold plated mirror and a 50W halogen bulb (Phillips) with an axial filament to emit a broadband NIR enhanced spectrum. The output spectrum is filtered to remove wavelengths below 504 nm and above 1068 nm to reduce UV exposure and heating effects. The optical intensity delivered to the tissue was 130 mW/cm^2^, which is within tissue safety limits for incoherent source of 200 mW/cm^2^ [33]. Each of the two light sources has a solenoid shutter (Melles Griot, USA) that is controlled by an electronic shutter controller (Melles Griot, USA). Light from each source is directed to the subject via a custom-built bifurcated optical fibre bundle (Fig. 1(b)) (individual glass fibres having diameter of 70 µm, NA= 0.57, Loptek Glasfasertechnik GmbH, Germany). The bundle diameter at the light source end is 4.5 mm, which then splits into two individual fibre bundles with 3.2mm diameter each (Fig. 1(b) and 1(c)) at the subject end. This effectively creates two pairs of source fibre bundles (Fig. 1(c)). A time multiplexing mechanism is used to control the open and close of the shutters of the light sources (Fig. 1(a)) hence allowing one light source to be on every 1.4 s, delivering light to the pair of source fibre bundles connected to it. As a result, a single complete acquisition cycle takes 2.8 s (or 0.35 Hz) and this allows data from all 24 measurement channels to be acquired.

To collect, separate and measure the intensity of light from the tissue at each wavelength, the system has two custom built spectrographs, each of which is connected to a front illuminated CCD camera (PIXIS: 512f, Princeton Instruments). The spectrograph is lens-based rather than mirror-based to provide higher light-throughput. The spectrograph contains Minolta MC Rokkor lenses with the focal length of f = 58 mm and an f-number of f/# = 1:1.2. The grating of the spectrograph (GR50-0310, Thorlabs) has dimensions of 50 x 50 x 9.5 mm with 300 grooves per mm blazed at 1000 nm to optimise reflection in the NIR region. The light spectrum is detected by two CCD cameras (PIXIS: 512f, Princeton Instruments) each with chip dimension of 12.3 x 12.3 mm corresponding to 512 x 512 pixels with a pixel size of 24 x 24 μm. The CCD cameras are cooled to −70 °C to reduce thermal noise and increase sensitivity. Each spectrograph is connected to a 3 m long detector fibre bundle (individual glass fibres having a diameter of 70 µm, NA = 0.54, Loptek Glasfasertechnik GmbH, Germany) (Fig. 1(d)), which at the subject end branches into six individual fibre bundles (Fig. 1(e)), five with bundle diameter of 1.5 mm and one with bundle diameter of 0.6 mm. Overall, at the subject end, the system has four source fibre bundles and 12 detector fibre bundles resulting in a total of 24 measurement channels.

Figure 2

**Fig. 2:**
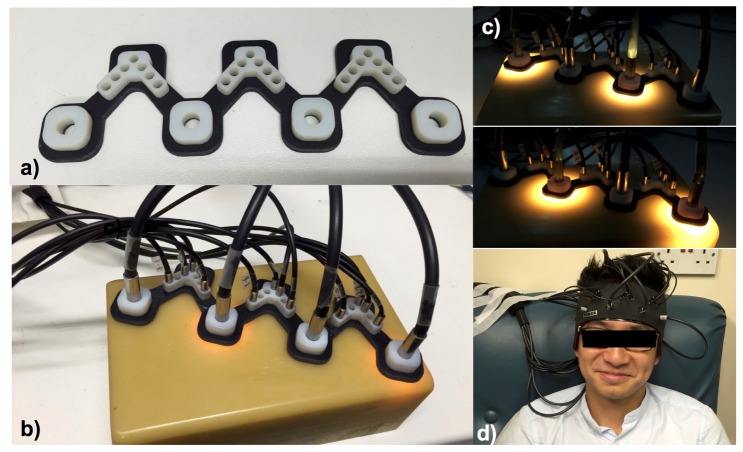
The interface between the system and the subject. (a) 3D printed optode holder. (b) Four source fibre bundles and 12 detector fibre bundles fixed on the optode holder on a solid phantom. (c) Illustration showing one complete acquisition cycle during which each of the two pairs of the source fibre bundles delivers light one after the other. (d) The optode holder with fibre bundles is fixed on a subject forehead using a headband.

shows the interface between the system and the subject. The optical interface between the optical fibre bundles and the subject is provided by an optode holder (Fig. 2(a)) that was designed specifically for the application on the adult forehead, enabling multi-channel NIRS measurements simultaneously. The holder was designed using AutoCad (AutoDesk) software to accommodate the dimensions of the fibre bundle heads and to ensure enough friction to hold the bundle heads in place during application (Fig. 2(b)). The holder was 3D printed with a combination of hard (Vero White) and flexible (Tango Black) materials to accommodate the curvature of the adult human forehead. Figure 2(c) demonstrates the operation of the system with the time multiplexing mechanism, switching light to the paired sources every 1.4 s. Figure 2(d) demonstrates the application of the system on a subject’s forehead.

**Fig. 3:**
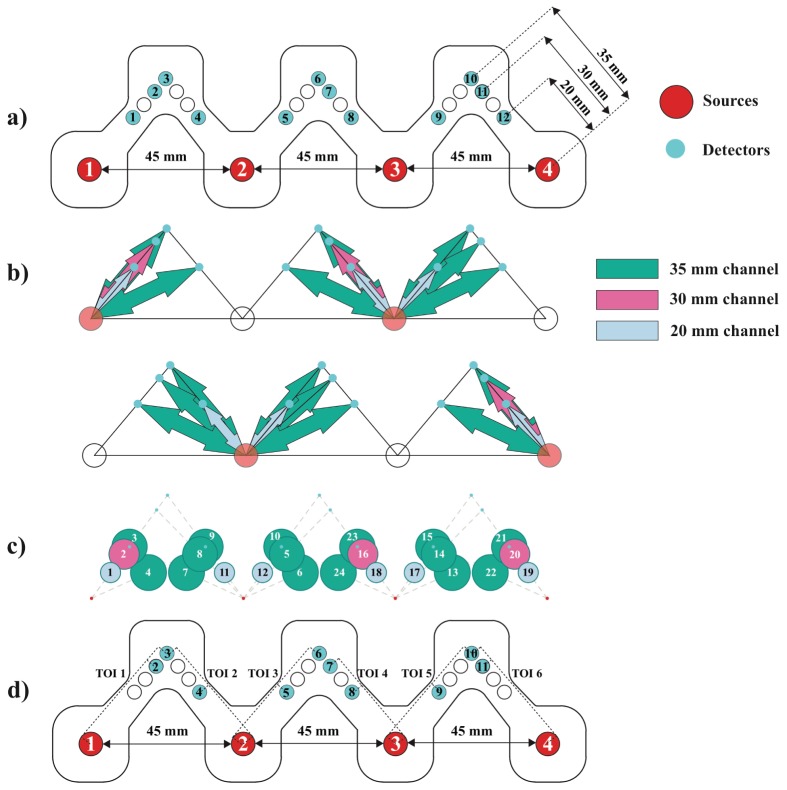
(a) Probe design showing the locations of four source fibre bundles numbered from 1 to 4 (red circles) and 12 detector fibre bundles numbered from 1 to 12 (cyan circles).The distance between neighbor source bundles is 45 mm and the SDS ranges from 20 mm to ~35 mm*. (b) Measurement channels acquired when alternative paired source bundles are on. Red circles indicate the locations of the working source bundles and cyan circles indicate the locations of the detector bundles. Dark green, magenta and light blue arrows indicate channels with SDS of 35 mm, 30 mm and 20 mm respectively. (c) The spatial distribution of all 24 measurement channels. Colour coding for SDS is similar to b. Circle size is proportional to SDS. (d) Detector bundles used for each TOI calculation. TOI 1 uses detector bundles 2 and 3, TOI 2 uses detector bundles 3 and 4, TOI 3 uses detector bundles 5 and 6, TOI 4 uses detector bundles 7 and 8, TOI 5 uses detector bundles 9 and 10 and TOI 6 uses detector bundles 10 and 11. *The absolute geometrical distances between source and detector ranges from 20mm to 35.6mm; however due to uncertainties introduced by the forehead curvature the actual source detector distance is most likely to be slightly different to the geometrical one.

The optode holder design has four source locations (numbered 1 to 4), with inter-source distance of 45 mm, and detector locations arranged to enable 24 broadband NIRS measurement channels and six TOI measurements using 12 detector fibre bundles (numbered 1 to 12) (Fig. 3(a)

). Four source fibre bundles and 12 detector fibre bundles were placed in the optode holder, as shown in Fig. 3(a), so that two 0.6 mm diameter detector fibre bundles occupy location 1 and 12 and the other locations were filled with 1.5 mm diameter detector fibre bundles. Detector fibre bundles from location 1 to 6 were from one common bundle connected to the first spectrograph, and detector fibre bundles from location 7 to 12 were from the other common bundle connected to the second spectrograph. The source-detector separations (SDS) of the 24 channels range from 20 mm to 35 mm. During operation, source fibre bundles are fixed in the holder such that the two source fibre bundles that are operating simultaneously have a separation of 90 mm (as demonstrated in Fig. 2(c)) ensuring that detector fibre bundles only receive light from one source fibre bundle at a time. Figure 3(b) shows measurement channels that are acquiring data when the paired sources are on, and Fig. 3(c) shows the spatial locations of all 24 possible measurement channels. Figure 3(d) shows the locations of detector fibre bundles that are used for the calculation of TOI that will be discussed in more detail in algorithm section. The optode holder is fixed on the subject’s forehead using double-sided medical tape laser-cut in the shape of the optode holder to reduce movement artefacts. Overall, the system has the capability of acquiring data from 24-channel NIRS measurements of changes in [HbO_2_], [HHb] and [oxCCO] and measurement of TOI from six regions of the forehead.

A LabVIEW program (National Instruments) was used to integrate light source switching with optical intensity measurement of the CCD cameras. The program removes the background thermal noise before combining intensity measurements of light from the detector bundle projected over different binned regions of the CCD camera. All data from every individual detector bundles connected to one spectrograph are captured at the same time. The intensity spectra from 504 to 1068 nm were projected over the CCD x axis and individual detector bundles were separated in the CCD y axis. To normalize the intensity spectrum measured by different detector bundles, gain factors for each of them were estimated. Reference intensity spectra from every detector bundles when they were exposed to the same amount of light input from an integrating sphere were recorded. Gain factors were then calculated by dividing the intensity spectrum of each of the 1.5 mm diameter bundles to the intensity spectrum of the 0.6 mm diameter bundle.

### 2.2 Algorithms

The UCLn algorithm (1) was used to convert the attenuation change measured across 108 wavelengths (from 780nm to 900nm) to concentration changes of chromophores applying the Modified Beer-Lambert Law (MBLL) [10, 30]. UCLn algorithm is a least-squares fitting procedure, finding the best fit of chromophore concentration changes based on the chromophores’ extinction coefficients and the measured attenuation changes over n number of wavelengths. A large number of wavelengths was used, as suggested by Matcher and colleagues [10], to resolve ∆[oxCCO] because calculating ∆[oxCCO] from a small number of wavelengths can give spurious changes.

$$\left(\begin{array}{l} \Delta [HbO_{2}]\\ \Delta [HHb]\\ \Delta [oxCCO] \end{array}\right)=\frac{1}{pathlength}{\left(\begin{array}{ccc} \begin{array}{l} \varepsilon_{HbO_{2}}(\lambda_{1})\\ \varepsilon_{HbO_{2}}(\lambda_{2}) \end{array} & \begin{array}{l} \varepsilon_{HHb}(\lambda_{1})\\ \varepsilon_{HHb}(\lambda_{2}) \end{array} & \begin{array}{l} \varepsilon_{oxCCO}(\lambda_{1})\\ \varepsilon_{oxCCO}(\lambda_{2}) \end{array}\\ \vdots & \vdots & \vdots \\ \varepsilon_{HbO_{2}}(\lambda_{n}) & \varepsilon_{HHb}(\lambda_{n}) & \varepsilon_{oxCCO}(\lambda_{n}) \end{array}\right)}^{-1}\left(\begin{array}{c} \Delta A(\lambda_{1})\\ \Delta A(\lambda_{2})\\ \vdots \\ \Delta A(\lambda_{n}) \end{array}\right).$$ (1)

The differential attenuation (ΔA) is calculated from measurements of the intensity spectrum. The optical pathlength used for the UCLn algorithm is the product between the source-detector separation and the differential pathlength factor (DPF). The DPF at 807nm was assumed to be 6.26 for all detectors [34]. The value of DPF for other wavelengths from 780 to 900 nm was calculated by taking into account the wavelength dependence of the DPF as DPF falls with increasing wavelength [16, 35].

TOI was calculated using the principle of SRS [12], an established technique based on measuring light attenuation at several different SDSs. Detailed description of the technique has been described elsewhere [12, 30]. Briefly, the attenuation slope (∂A/ ∂ρ) was calculated using the intensity measurements from two detectors at different distances for each wavelength from 740-900 nm. The result was used to calculate the scaled absorption coefficient spectrum kμ_a_ (k is an unknown constant), taking into account the wavelength dependency of scattering by applying a correcting factor (1-hλ) where h = 0.00085 mm^−1^nm^−1^ [36]. The kμ_a_ was then used to calculate the scaled absolute concentrations of HbO_2_ (k[HbO_2_]), HHb (k[HHb]) and water (k[H_2_O]). The scaled concentrations were used to calculate TOI, the ratio between k[HbO_2_] and scaled total haemoglobin k([HbO_2_] + [HHb]).

### 2.3 Study protocol and data analysis

#### 2.3.1 Stability and noise characterisation

In order to investigate the thermal noise and dark count of the CCD cameras, data were acquired from all 12 detector fibre bundles for 20 minutes with the shutter of the spectrograph closed. Intensity distribution across 108 wavelengths and the change in intensity of individual wavelength along time were investigated.

#### 2.3.2 Source interference

Given that the system utilises two light source bundles at the same time there is a risk that one detector bundle will receive light from both of the source bundles. To characterise this possible interference, two experiments were performed, one of which was on a homogeneous phantom with tissue-like optical properties and the other on a healthy adult volunteer forehead. The optode holder was fixed on the phantom or the forehead using double-sided tape and 12 detector fibre bundles were positioned on the holder as described in Fig. 3(a) to collect the intensity data. One source fibre bundle was translated consecutively from source locations 1 to 4. To represent the intensity measured by each detector bundle, mean intensity across 108 wavelengths from 780 to 900 nm was calculated for all 12 detector bundles for both experiments. Using data from individual source illumination, SNR was calculated for 24 channels to assess and quantify the effect of double illumination. SNR is defined as the ratio of intensity count used for the measurements and the intensity due to noise. The SNRs when sources 1 and 3 were on and when sources 2 and 4 were on were calculated.

#### 2.3.3 Frontal lobe functional activation

Following ethics approval and volunteer consent, a pilot study was conducted in nine healthy adult volunteers with the probe holder positioned across the midline of the forehead such that source locations 2 and 3 were located at Fp2 and Fp1 according to 10/20 EEG electrode placing system [37]. Prefrontal lobe activation was achieved with a modified version of the Stroop task [38]. The block design consisted of 10 epochs, each with a 30 s black screen followed by a 60 s Stroop task and then a 30 s black screen. Total acquisition time was 1200 s. During the 60 s Stroop task, subjects were presented with a series of coloured words written in coloured ink and asked to name the colour of the individual word while ignoring its meaning. Four colour options were used: red, green, blue and magenta. The stimulation was presented to the subject using a 17-inch laptop computer positioned 40 cm away from the subject and the subject responded by pressing the corresponding keys. Attenuation changes were acquired by 12 detector fibre bundles continuously while the light from the paired source fibre bundles was switching. Time course concentration changes of the three chromophores (∆[HbO_2_], ∆[HHb] and ∆[oxCCO]) were calculated from the acquired attenuation changes using the UCLn algorithm as described previously. Concentration changes were filtered using a 5th order Butterworth low pass filter with cut-off frequency of 0.0875 Hz, linearly detrended and then resampled to 1 Hz. For each subject, concentration changes were block-averaged across each epoch to produce three 120s traces (∆[HbO_2_], ∆[HHb] and ∆[oxCCO]) for each measurement channel. The mean concentration changes in individual channels were then averaged across nine subjects to produce the grand average activation map across the frontal lobe for the Stroop task. In this study, cortical activation is defined by an increase in Δ[HbO_2_], decrease in Δ[HHb] and increase in Δ[oxCCO]. To identify channels with significant changes due to functional stimulation, the response of each channel for each subject was defined as the difference between the mean of the first 10s window of activation and the mean of the first 10s window during the black screen period. Paired t-tests were used to compare the responses of each channel across all nine subjects to zero (i.e no response). p < 0.05 was considered statistically significant. This was done for each of the three chromophores.

#### 2.3.4 Tissue oxygen saturation

TOI values were calculated as described earlier for six sites of measurements indicated in Fig. 3(d) as TOI 1 to TOI 6. Using the slope of the attenuation between the closest detector (20 mm SDS) and the furthest detector (35 mm SDS) for sites 2, 3 and 5, between the 30 mm and the 35 mm detectors for sites 1 and 6, and between the 20 mm and the 30 mm detectors for site 4, scaled absorption coefficients (kμ_a_) were calculated and then used to estimate scaled absolute concentrations of HbO_2_ and HHb (k[HbO_2_] and k[HHb]) for each site. These values were then used to calculate TOI for different sites [30]. To investigate the spatial variations in TOI in different regions, mean TOI was calculated by taking the average of TOI values across the whole experiment period for each of the six sites.

#### 2.3.5 Systemic data analysis

Systemic physiological monitoring data were collected simultaneously with NIRS measurements. Arterial blood pressure (ABP) was measured with a Portapres® system (Finapres Medical Systems, the Netherlands); scalp blood flow was measured by Laser Doppler Monitor (Moor Instruments); and end-tidal carbon dioxide (EtCO_2_), arterial oxygen saturation and heart rate were measured with a clinical monitor IntelliVue (Philips, the Netherlands). Systemic changes were block-averaged according to epoch and averaged across nine subjects. For each systemic variable, the response for each subject was defined as the difference between the mean of the first 10s window of activation and the mean of the first 10s window during the black screen period. Paired t-tests were used to compare the responses across nine subjects to zero, identical to the statistical analysis done for the NIRS signals.

## 3. Results

### 3.1 System stability and noise characterisation

**Fig. 4:**
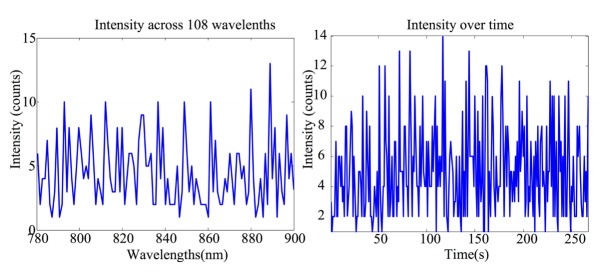
(a) The intensity spectrum measured by one detector fibre bundle when the shutter to the spectrograph is closed. (b) The intensity change at wavelength 780 nm over time during the test.

The intensity measurement across 108 wavelengths (from 780nm to 900nm) of one detector fibre bundle is presented in Fig. 4(a)

. The distribution has a flat shape with no prominent features and low intensity value as expected when there is no light input into the spectrograph. Figure 4(b) shows the intensity change at one wavelength over time (λ = 780nm). The intensity change has a mean value of 5 ± 3 counts.

### 3.2 Source interference and channel selection for analysis

Table 2

**Table 2:** Intensity counts from 12 detector fibre bundles with individual sources on the phantom.

Detector number	**Source 1**	**Source 2**	**Source 3**	**Source 4**	**1 and 3 SNR**	**2 and 4 SNR**
**1**	776	963	16	9	48.50	107.00
**2**	2793	4240	32	8	87.28	530.00
**3**	1032	4650	43	8	24.00	581.25
**4**	144	13150	15	8	9.60	1643.75
**5**	21	10858	58	8	**2.76**	1357.25
**6**	103	4841	784	10	7.61	484.10
**7**	8	2640	4879	182	609.88	14.51
**8**	4	162	4693	20	1173.25	8.10
**9**	4	21	6482	227	1620.50	10.81
**10**	7	129	1910	1991	272.86	15.43
**11**	6	8	1759	4499	293.17	45.91
**12**	6	30	361	119	60.17	**3.97**

and Table 3

**Table 3:** Intensity counts from 12 detector fibre bundles with individual source on an adult forehead

Detector number	**Source 1**	**Source 2**	**Source 3**	**Source 4**	**1 and 3 SNR**	**2 and 4 SNR**
**1**	490	505	7	5	70.00	101.00
**2**	2506	2402	12	4	208.83	600.50
**3**	956	2543	16	5	59.75	508.60
**4**	120	8655	5	4	24.00	2163.75
**5**	9	4636	32	4	**3.56**	1159.00
**6**	57	2584	421	6	7.39	430.67
**7**	7	1621	2737	76	391.00	21.33
**8**	4	98	2601	8	650.25	12.25
**9**	5	8	3210	143	642.00	17.88
**10**	5	53	1018	1279	203.60	24.13
**11**	4	42	948	3098	237.00	73.76
**12**	5	14	175	45	35.00	**3.21**

present the intensity values collected by 12 detector fibre bundles (numbered according to Fig. 3(a)) on the homogeneous phantom and on the adult forehead respectively. These are intensity values when only one light source fibre bundle was used at location 1 to 4 successively. The shaded grey values on each column are from detector fibre bundles that are used for measurement channels for each of the light source bundle as indicated by Fig. 3(b). The non-shaded counts are from the detector bundles that are not used for measurement channels. 1 and 3 SNR was calculated by dividing the intensity values from the shaded column to the non-shaded one using column 1 and 3 only. Similar calculations were used for 2 and 4 SNR. There are two cases where SNRs are small (<5) (indicated by bold numbers in Table 2 and 3) in both the phantom and adult head. The first happens between source 3 and detector bundle 5, corresponding to channel 24 and the second happens between source 4 and detector bundle 12, corresponding to channel 19. Those two channels are excluded from further analysis.

### 3.3 Functional activation

**Fig. 5:**
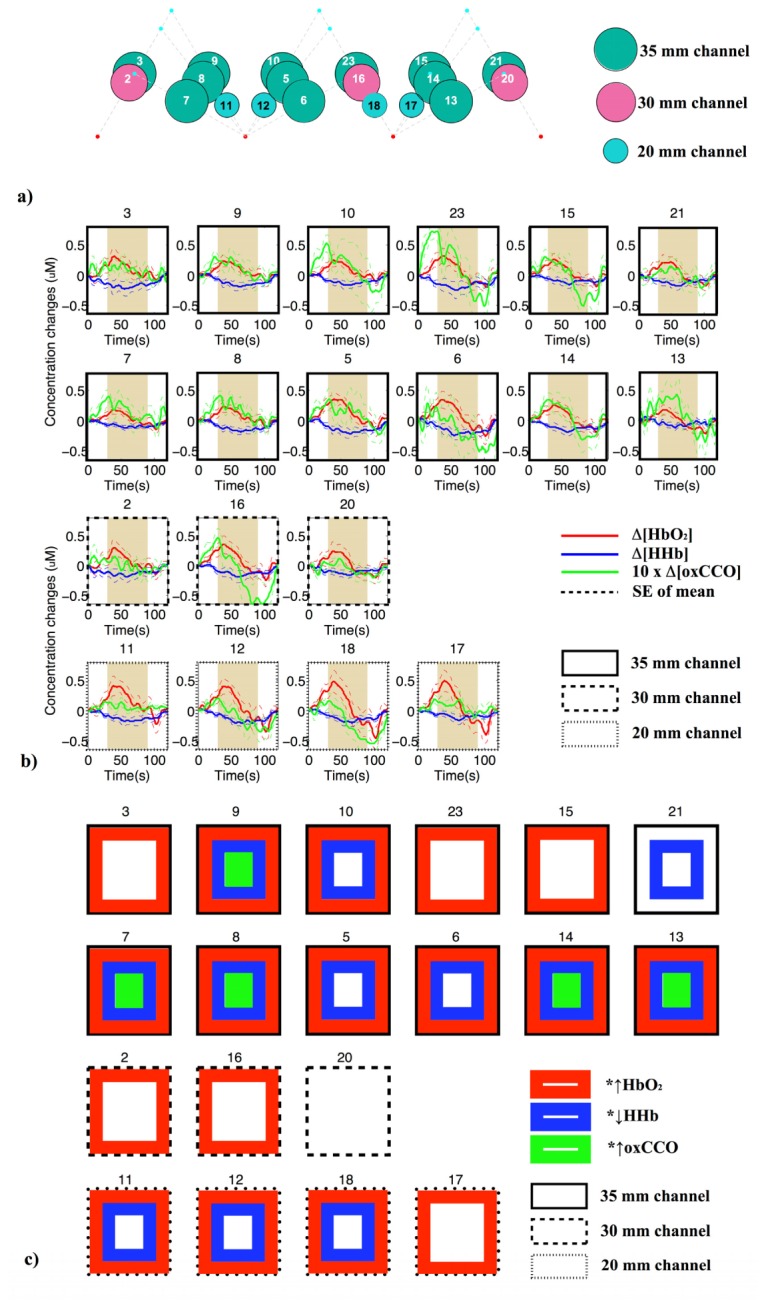
(a) Positions of 19 NIRS measurement channels on the adult forehead. Dark green, magenta and light blue circles indicate channels with SDS of 35 mm, 30 mm and 20 mm respectively. (b) Grand average concentration changes and standard error of mean of HbO_2_ (red line), HHb (blue line) and 10 x (oxCCO) (green line) among 19 channels across nine subjects during the Stroop task. Plots with solid black borders are from channels with 35 mm separation, dashed black are from channels with 30 mm and dotted ones are from channels with 20 mm separation. The 60 s activation period is indicated by the shaded area. (c) Channels with significant increase in ∆[HbO_2_] (red squared border), decrease in ∆[HHb] (blue squared border) and increase in ∆[oxCCO] (green squared border).

Based on the values of the intensity collected from different channels using the experimental system described in the Methods section, channels 1, 4 and 22 (Fig. 3(c)) are excluded from analysis due to low intensity. Figure 5(a)

presents the positions of 19 channels on the forehead. Figure 5(b) presents group block-averaged concentration changes and standard error of mean of the three chromophores during the Stroop task across nine subjects with different SDS indicated by the borders of the plots. The concentration change of oxCCO is multiplied by 10 for ease of visualisation. There is a spatial variation of the oxCCO response to frontal lobe activation among channels with 35 mm SDS induced by the Stroop task with maximum changes in ∆[oxCCO] varying from +0.015 µM (channel 21) to +0.073 µM (channel 23) in the presence of haemodynamic changes correlating to functional activation (increase in ∆[HbO_2_], decrease in ∆[HHb]). Figure 5(c) presents locations of the channels with significant response in ∆[HbO_2_], ∆[HHb] and ∆[oxCCO]. Out of 12 channels with SDS of 35 mm, 11 channels (92%) show significant increase in ∆[HbO_2_]; nine channels (75%) show significant decrease in ∆[HHb] and five channels (42%) show significant increase in ∆[oxCCO]. Significant change in ∆[oxCCO] was only present in channels where there were significant changes in both ∆[HbO_2_], ∆[HHb]. Significant change in ∆[oxCCO] was not present in 30 mm or 20 mm SDS channels. However, significant changes in ∆[HbO_2_] were present in the 30 mm SDS (two out of three channels) and in 20 mm SDS (three out of four channels). Table 4

**Table 4:** Mean response (Resp.) across nine subjects for each chromophore and the corresponding p value in channels with significant oxCCO changes.

	**Channel 7**		**Channel 8**		**Channel 9**		**Channel 13**		**Channel 14**	
	**Resp. (µM)**	**p**	**Resp. (µM)**	**p**	**Resp. (µM)**	**p**	**Resp. (µM)**	**p**	**Resp. (µM)**	**p**
**∆[HbO_2_]**	0.135	0.026	0.176	0.018	0.185	0.022	0.163	0.033	0.193	0.026
**∆[HHb]**	−0.059	0.007	−0.116	0.005	−0.110	0.014	−0.060	0.032	−0.093	0.020
**∆[oxCCO]**	0.024	0.005	0.033	0.009	0.024	0.036	0.028	0.027	0.029	0.035

presents the mean response across nine subjects for ∆[HbO_2_], ∆[HHb] and ∆[oxCCO] and the corresponding p value in channels with significant ∆[oxCCO] change as indicated in Fig. 5(c) (an extended table with the mean responses and p values for all channels is included as supplementary Table 1).

### 3.4 Tissue oxygen saturation

There is no observable TOI response during functional activation. Table 5

**Table 5:** TOI (%) across 6 measurement sites of nine subjects

Subject number	**Site 1**	**Site 2**	**Site 3**	**Site 4**	**Site 5**	**Site 6**	**Mean**	**SD**
**1**	79	73	74	77	80	73	76	3
**2**	81	73	70	72	76	N/A	74	4
**3**	65	69	67	69	70	66	68	2
**4**	89	74	73	78	80	87	80	6
**5**	66	70	70	73	73	69	70	2
**6**	78	74	75	73	77	76	76	2
**7**	81	73	76	82	76	83	78	4
**8**	61	69	69	71	72	75	69	4
**9**	79	76	78	77	79	73	77	2
**Mean**	76	72	72	75	76	75		
**SD**	9	2	4	4	4	6		
**Min**	61	69	67	69	70	66		
**Max**	89	76	78	82	80	87		

presents the mean TOI values during the experiment across six sites of measurement on the frontal lobe for each individual subject and the mean, standard deviation (SD), maximum and minimum values of TOI across nine subjects.

### 3.5 Multimodal data analysis

**Fig. 6:**
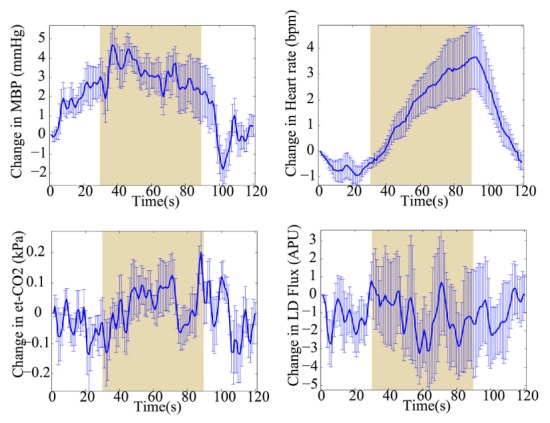
Grand average changes in systemic variables (mean ± standard error) during frontal lobe functional activation induced by Stroop task. (a) Change in mean blood pressure (mmHg). (b) Change in heart rate (bpm). (c) Change in end tidal carbon dioxide (kPa). (d) Change in scalp blood flow in arbitrary Perfusion Unit. The shaded areas indicate the 60 s stimulation period.

There were significant differences (p<0.05) in the change in the mean blood pressure during functional activation induced by the Stroop task with the mean ± SD change of 2.7 ± 1.9 mmHg. There was no significant difference in the end tidal carbon dioxide, arterial oxygen saturation, or scalp blood flow during the functional activation task. Regarding the change in heart rate, there was no significant change during the first 10 s of stimulation but there was a significant change during the last 10 s of stimulation compared to the baseline (p<0.05). Figure 6

shows grand average changes of systemic variables across subjects including a) mean blood pressure, b) heart rate, c) end-tidal CO_2_ and d) laser Doppler scalp blood flow. Variation in saturation is negligible.

## 4. Discussion

We have developed a multi-channel, multi-distance broadband near-infrared spectroscopy system with the capability of investigating the haemodynamic and oxCCO responses at multiple distances and locations across the prefrontal cortex. The system also has the capability of monitoring tissue oxygen saturation at multiple locations. Compared to previously described systems (as summarized in Table 1), our system can record ∆[HbO_2_], ∆[HHb] and ∆[oxCCO] from 24 simultaneously measured channels and TOI from six regions - by far the highest number of channels/regions for these measurements reported to date. We used the system to record functional activation-induced changes of ∆[HbO_2_], ∆[HHb] and ∆[oxCCO] in 19 channels in nine healthy adult volunteers across the prefrontal cortex and identified channels with significant changes in ∆[HbO_2_], ∆[HHb] and ∆[oxCCO]. Simultaneous with changes in these NIRS variables, we have also demonstrated that TOI differs slightly across different sites in the prefrontal cortex and across subjects. In addition, our system can be used in conjunction with systemic monitoring devices to acquire datasets including blood pressure changes, scalp blood flow changes, end tidal carbon dioxide, arterial oxygen saturation and heart rate. These datasets can provide a platform to investigate the spatial response of ∆[oxCCO] during functional activation in the presence of typical haemodynamic changes. Moreover, confounding effects of systemic physiology changes on NIRS functional activation data resulting in false positive or false negative interpretation of NIRS result [39] can also be investigated.

System stability tests show that the dark noise of the system is randomly distributed across 108 wavelengths of measurements with a relatively low photon counts. Intensity measured over time shows an expected small variation due to system noise.

The source interference test indicates that most of the measurement channels used for the calculation of concentration changes during the functional activation task have high SNRs; two channels did not and these were excluded from further analysis. The values of SNRs are relatively similar in both the phantom and the adult forehead; however, in general the SNRs from the adult forehead are more evenly distributed among measurement channels. This method of SNR calculation can be used in future experiments to characterise the behavior of each measurement channel before any experimental protocol. The differences observed in the light intensity measured by different detectors on the phantom having the same SDS may be due to a combinations of factors. These might include differences (i) in the diameter of detector fibre bundles (1.5 mm and 0.6 mm) and (ii) in the amount of light entering the spectrographs due to small differences in slit width between spectrographs. However, these factors do not change over time. As a result, the attenuation measured by each channel can be reliably used to resolve concentration changes of chromophores.

The maximum and minimum TOI values for six sites were within the range from 61% to 89%. This range of TOI values agrees with the range of baseline TOIs from a large study performed by Al Rawi and colleagues to investigate the anatomical source of the calculated values of TOI by SRS technique in the adult heads using a NIRO 300 (Hamamatsu Photonics) during the clamping of internal and external carotid arteries [13]. This study provides evidence to support the hypothesis that TOI measurement reflects changes in cerebral tissue oxygenation. In our study there were small variations in TOI values across different sites within the same subject, and small variations of TOI values within the same site across different subjects. TOI does not have sufficient SNR to detect the oxygenation changes due to functional activation (this is an effect of the use of the slope to estimate the scaled absorption). The measurement of TOI across different brain regions may be used to assess absolute tissue oxygen saturation across these regions for clinical applications.

Considering the NIRS result from this frontal lobe activation study, the group data shows changes of ∆[HbO_2_] and ∆[HHb] that are consistent with typical functional activation with increases in ∆[HbO_2_] and decreases in ∆[HHb] during the stimulation period. However, during the 60s stimulation period, ∆[HbO_2_] increases initially for around 10s then reaches a plateau before declining rapidly below the baseline. This finding agrees with a study using a similar Stroop protocol to investigate the changes in concentration of haemoglobin signals in different prefrontal cortex areas [40]. We have observed a small and insignificant difference in the magnitude of the ∆[HbO_2_] response between different SDS (see supplementary Table 1). Short SDS measurements are confounded by the extracranial circulation and are largely affected by systemic changes and anxiety levels [41, 42]. Therefore, in certain circumstances large haemoglobin changes can be seen in short SDS channels. This makes it difficult to predict the magnitude of the response in the short channel and compares it to that from the longer SDS channels that sample both a portion of the extracranial and intracranial circulations. This is one of the rationales to investigate the possibility of using oxCCO signal for functional brain imaging to identify the specific location of the functional activation as oxCCO signal is less susceptible to changes in systemic variables and more brain specific [30]. Regarding the oxCCO response in this study, the depth dependency characteristic can be seen when comparing the response in paired channels sampling the same/similar tissue but at different depths. For example, comparing oxCCO response in the short (20 mm) and the long channels (35 mm), such as channels 11 and 9, or channel 12 and 10, as well as 18 and 23, 17 and 15, a bigger maximum magnitude change in the channel with longer SDS can be observed. Such depth-dependency response of the oxCCO signal, reflecting its brain specificity characteristic, has been reported previously by Kolyva and colleagues [30].

Comparing the spatial variation of the oxCCO response to the haemoglobin responses (Fig. 5(c)), a significant change in Δ[oxCCO] was only seen in five channels with 35 mm SDS across the forehead where there were concurrent significant change in both ∆[HbO_2_] and ∆[HHb]. In contrast, significant changes in Δ[HbO_2_] and Δ[HHb] were seen in most of the 35 mm SDS channels (92% and 75% respectively), some of the 30 mm channels (67% and 0% respectively) and most of the 20 mm channels (100% and 75% respectively). The Stroop task is known to activate bilateral dorsolateral prefrontal cortices (DLPFC) [43, 44]. As we have explicitly stated in the paper, we consider activation as a significant increase in Δ[HbO_2_], a significant decrease in Δ[HHb] and a significant increase in Δ[oxCCO]. With our array positioned on the prefrontal cortex where source locations 2 and 3 were positioned at Fp2 and Fp1 of the 10/20 EEG electrode placement system, our results identify bilateral activation of the DLPFC. The haemoglobin signals are known to be confounded by extracranial contamination [39], while the oxCCO signal is more depth resolved [30, 45]. We can identify a more localised activation area when the oxCCO responses are considered in addition to HbO_2_ and HHb. The increase in oxygen consumption is previously suggested to be more localised than the increase in blood flow [46] and our data might suggest that. Larger studies are required to differentiate regional variation in the magnitude of oxCCO response.

There were also changes in some systemic physiological variables during the Stroop task. The significant change in blood pressure, although small in magnitude, may confound the NIRS results leading to false-positive or false-negative responses as described by Tachtsidis and Scholkmann [39].

To our knowledge, this is the first study demonstrating the difference in oxCCO response to functional activation across multiple areas of the frontal cortex that are distinct from the haemoglobin responses. Previously, the spatial distribution of oxCCO response to functional activation has been measured in the visual cortex using similar setup [47]; however, the acquisition time using that system was slow and the recordings of all measurement channels were not collected simultaneously. Using the multi-channel broadband NIRS system described in this paper, multi-channel measurements of changes in haemodynamics and metabolism as well as tissue oxygen saturation can be acquired simultaneously. This multi-channel, multi-wavelength data can be used to reconstruct images of ∆[HbO_2_], ∆[HHb] and especially ∆[oxCCO] which may open up a new frontier to investigate cellular oxygen metabolism across multiple areas of the human cortex in different clinical scenarios.

## 5. Conclusion

We have developed a multi-channel, multi-distance broadband NIRS system to measure not only changes in haemodynamics (∆[HbO_2_] and ∆[HHb]), but also changes in cellular oxygen metabolism (∆[oxCCO]) and absolute tissue oxygen saturation (TOI) at multiple locations across the human adult prefrontal cortex. We have used the new system to collect functional NIRS data across the prefrontal cortex of nine healthy adult volunteers during a Stroop task and identified channels with significant change in Δ[HbO_2_], Δ[HHb] and Δ[oxCCO]. Multi-channel, multi-distance NIRS datasets collected in conjunction with systemic data allow multimodal data analysis. This provides an unprecedented opportunity to understand and interpret the spatial response of NIRS variables in multiple locations, especially the NIRS-derived oxCCO signal, during functional brain activation.
